# Chromatin‐modifying drugs and metabolites in cell fate control

**DOI:** 10.1111/cpr.12898

**Published:** 2020-09-26

**Authors:** Ziyue Yao, Yu Chen, Wenhua Cao, Ng Shyh‐Chang

**Affiliations:** ^1^ State Key Laboratory of Stem Cell and Reproductive Biology Institute of Zoology Chinese Academy of Sciences Beijing China; ^2^ Institute for Stem Cell and Regeneration Chinese Academy of Sciences Beijing China; ^3^ University of Chinese Academy of Sciences Beijing China

**Keywords:** acetylation, chromatin, DNA, histone, metabolism, methylation

## Abstract

For multicellular organisms, it is essential to produce a variety of specialized cells to perform a dazzling panoply of functions. Chromatin plays a vital role in determining cellular identities, and it dynamically regulates gene expression in response to changing nutrient metabolism and environmental conditions. Intermediates produced by cellular metabolic pathways are used as cofactors or substrates for chromatin modification. Drug analogues of metabolites that regulate chromatin‐modifying enzyme reactions can also regulate cell fate by adjusting chromatin organization. In recent years, there have been many studies about how chromatin‐modifying drug molecules or metabolites can interact with chromatin to regulate cell fate. In this review, we systematically discuss how DNA and histone‐modifying molecules alter cell fate by regulating chromatin conformation and propose a mechanistic model that explains the process of cell fate transitions in a concise and qualitative manner.

## Graphical abstract

Summary of DNA and histone‐modifying drugs. (a) DNA drugs mainly include three types: covalent binding agents, groove binders and insertion binders. (b) Histone drugs. Histone acetylation can be drugged via two families of proteins: HDACs and HATs. Amongst the HDAC inhibitors (HDACi), hydroxamates are the most widely used. Hydroxamates, such as TSA, panobinostat and SAHA, mainly function through the non‐covalent chelation of Zn^2+^ at the active site of HDACs. Together with the metabolic regulation of HATs, HDACi promote histone acetylation, eviction of histone H1 and nucleosome loosening. Amongst the HAT inhibitors (HATi), the most prominent are the natural compounds garcinol and curcumin, and the synthetic drug C646. Together with the metabolic regulation of HDACs, HATi promote histone deacetylation and tightly bound nucleosomes.

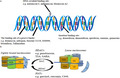

## 1 INTRODUCTION

Stem cells possess the potential for self‐renewal and differentiation. Based on this characteristic, stem cells have shown extraordinary potential in the treatment of cardiovascular diseases, neurodegenerative diseases, spinal cord injury, musculoskeletal diseases, diabetes and various organ failure syndromes.^1^, ^2^, ^3^, ^4^, ^5^, ^6^ Therefore, clarifying the mechanism of stem cell fate determination is extremely important for fulfilling the promise of regenerative medicine.

In this regard, the traditional view holds that transcription factors play a decisive role. Early research, such as the ectopic expression of *MyoD* and the more recent reprogramming of induced pluripotent stem cells (iPSCs) with *Oct4, Sox2, Klf4 and c‐Myc,* has confirmed the importance of transcription factors.^7^, ^8^ However, subsequent investigations found that transcription factors were insufficient in many cases, and there exists evidence of epigenetic memory or incomplete reprogramming, implying that transcription factors are not always the only factors determining cell fate.^9^, ^10^ The widespread changes in epigenetic modifications during cell fate transitions suggest that epigenetics may be another important dimension to consider. Epigenetic modifications, including DNA modifications and histone modifications,^11^, ^12^, ^13^ often lead to changes in chromatin conformation and sculpt the milieu for transcription factors to function.^14^ In addition, some studies have found that transcription factors also regulate the epigenetic properties near target genes by recruiting transcription coactivators, such as the histone acetyltransferase p300.^15^ Therefore, it appears that the interactions between epigenetic modifications and transcription factors regulate the conformation of chromatin, ie the 3D organization of the genome, to determine the fate of cells, through an as yet incompletely understood process.

These findings have promoted a general interest in the study of chromatin modifications and regulation. In recent years, some chromatin‐modifying drugs and metabolites have been shown to possess the ability to change the fate of cells,^16^, ^17^ but there is a lack of systematic synthesis of these myriad findings. In this review, we summarize the epigenetic effects of these small molecules, discuss the mechanisms of interactions between epigenetic regulation and transcription factors during chromatin changes in cell fate determination and hypothesize the potential value of these drugs.

## 2 THE RELATIONSHIP BETWEEN CHROMATIN AND CELL FATE

Stem cells have the unique abilities of long‐term self‐renewal and multipotent differentiation, which are essential for maintaining the stem cell population and tissue integrity. Since stem cells and their differentiated progeny share the same genome and differ only in their chromatin organization, increasing evidence suggests that the unique characteristics of stem cells are largely determined by chromatin patterns.^8^, ^18^, ^19^ The chromatin structure, dynamics and functions of stem cells are distinct from differentiated cells.^20^, ^21^, ^22^ For example, pluripotent stem cells have more open and easily accessible chromatin,^23^ which makes them highly plastic in their cell fate trajectories.

**Figure 1:**
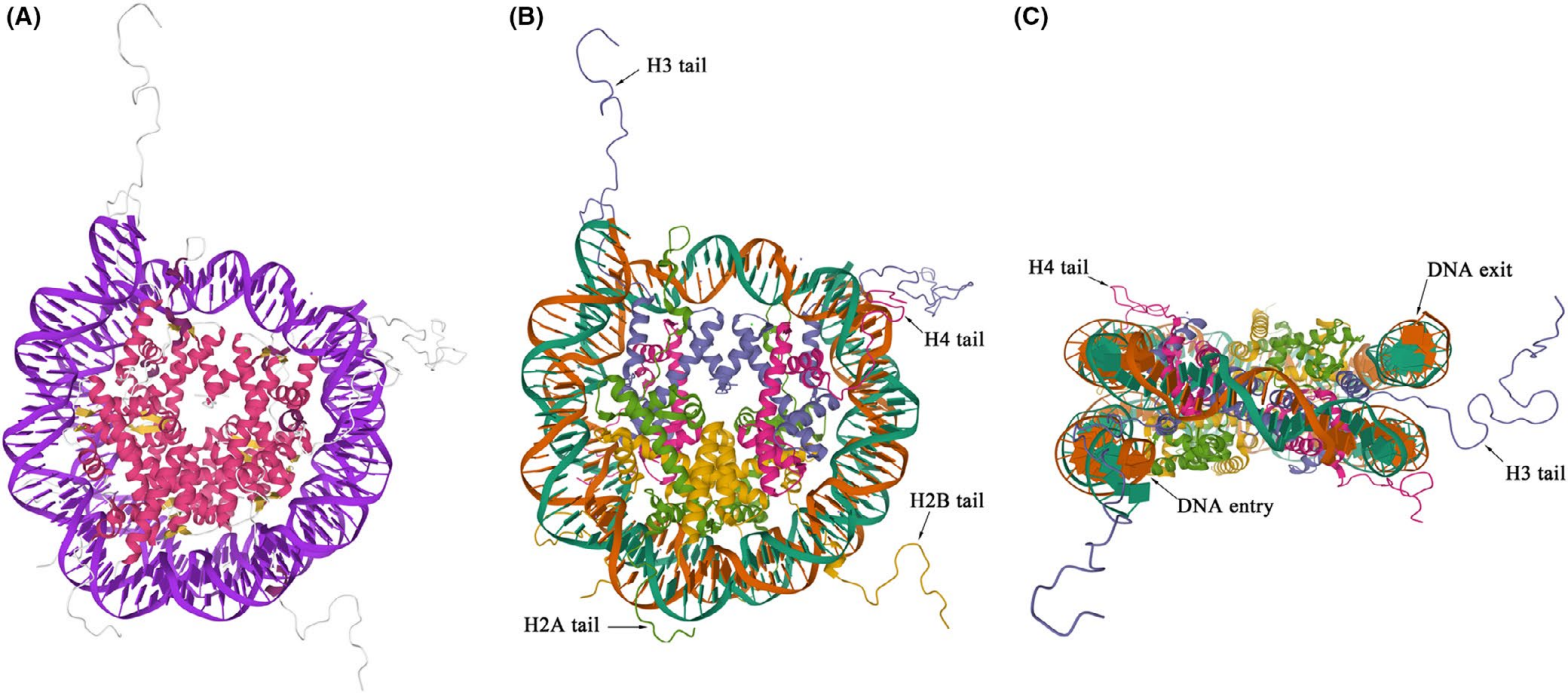
The 3D structure of the nucleosome (PDB code 1KX5).^27^ A, Top‐down view of the nucleosome with acidic DNA (blue) wrapped around histones with α‐helices (red) rich in basic residues. B, Top‐down view of the DNA double helix (green and brown) wrapped around the histone octamer core consisting of pairs of H2A (green), H2B (yellow), H3 (purple) and H4 (red), and their respective histone tails. C, Side view of the ~ 1.75 turns of DNA wrapped around the histone octamer core, with histone H3/H4 tails flanking the DNA entry/exit regions

The chromatin of eukaryotes is highly complex, with different levels of assembly structure and a compression ratio of up to 10 000. The nucleosome is the basic unit of chromosomes, which consists of two copies of two heterodimers H2A/H2B and H3/H4 to form a histone octamer (Figure 1), surrounded by double‐stranded DNA of about 146 bp.^24^ Histone subunits are rich in α‐helices with basic Arg and Lys residues, thus endowing them with net positive charges. This allows them to interact with the acidic and negatively charged DNA molecules, via ionic and hydrogen bonding. For example, the amino acid side chains of histone residues, such as H3R42 and H3T45, form hydrogen bonds with the oxygens in the phosphodiesters of DNA.^25^ The binding of DNA at the nucleosome entry/exit region (ie the head and tail of the DNA wrapped around the nucleosome) is not stable, but the internal DNA region near the bipartite axis is most tightly wrapped around the histones.^26^ The structural characteristics of nucleosomes mean the DNA entry/exit regions can easily unwind from histones, thereby initiating DNA replication, transcription and repair activities.

The Arg and Lys residues in histone subunits are not only critical for interactions with DNA, but also provide side chains amenable to chemical modifications that regulate chromatin structure and gene transcription. Histone methylation usually occurs in specific Arg and Lys residues of the histone tails,^28^ which have different effects on gene activity depending on the specific residues that are modified and the degree of methylation. Each Lys (K) residue has three possible methylation states: mono‐, di‐ or tri‐methylation. The di‐or tri‐methylation at H3K4, H3K36 and H3K79 is usually associated with transcriptional activation,^29^, ^30^, ^31^ while H3K9 and H3K27 methylation is generally associated with transcriptional repression.^29^, ^31^ H3K9me3 is a feature of heterochromatin,^32^, ^33^, ^34^, ^35^ while H3K9me2 is more common in silent or near‐silent genes of euchromatin.^34^, ^35^ In embryonic stem cells (ESCs), H3K9me3‐marked heterochromatin domains increase with differentiation, thus contributing to lineage restriction and cell fate determination.^32^

Stem cells also have chromatin domains with a special histone modification pattern called ‘bivalent domains’, which consist of large regions of repressive H3K27 methylation harbouring smaller regions of activating H3K4 methylation.^36^ Bivalent domains largely mark silent lineage‐specific genes that are ready to be activated at any time, so they can be quickly activated or repressed during differentiation and development.^37^ The bivalent domains in ESCs are regulated by BAF60 chromatin remodelling proteins, which regulate the redistribution of H3K4me3 and H3K27me3 and thus pluripotency.^38^ In addition to ESCs, some adult stem cells also have bivalent domains, such as hematopoietic stem cells and muscle stem cells.^39^, ^40^ During the early stages of muscle stem cell activation, bivalent domains increase via the expansion of the repressive H3K27me3 mark. When muscle stem cells commit to the myoblast stage, most of the bivalent domains resolve, and most genes in myoblasts revert to a monovalent state.^40^

Histone acetylation is often higher in undifferentiated ESCs and muscle stem cells than their differentiated progeny, and histone acetylation is known to control the dynamics of normal chromatin.^41^, ^42^, ^43^ Meanwhile, histone deacetylase inhibitors can significantly increase histone acetylation, thereby increasing the turnover dynamics of euchromatin proteins in mouse embryonic fibroblasts. Studies have also shown that histone deacetylase inhibition can promote the reprogramming of somatic cells into pluripotent cells and also help maintain ESCs in an undifferentiated state.^44^, ^45^ Thus, histone acetylation is closely related to the highly dynamic euchromatin, cellular plasticity and cell fate determination. Besides histone acetylation and methylation, other post‐translational modifications (PTMs) of histone tails may also affect the stability of the nucleosome core and its accessibility to chromatin remodelling complexes and DNA sequence‐specific transcription factors.^46^, ^47^, ^48^, ^49^ Most of these histone modifications require metabolic intermediates as their cofactors or coenzymes and are heavily influenced by the metabolic state of cells.

In addition, alterations in DNA topology also regulate the structure and function of chromatin by affecting the binding of DNA to nucleosomes.^50^, ^51^, ^52^ For example, DNA intercalator drugs such as doxorubicin can be inserted directly into the DNA double helix, thereby affecting the interactions between DNA and histones, resulting in changes in the 3D structure and function of chromatin.^52^ We will systematically review the role of metabolites and drugs in chromatin biology and formulate a unified model of how these small molecules might regulate cell fate.

## 3 CHROMATIN‐MODIFYING METABOLITES IN CELL FATE CONTROL

Histone tails play an important role in nucleosome stability,^53^ nucleosome localization^54^ and the binding of transcription factors to DNA,^55^, ^56^ In addition, probably because of their accessibility, histone tails are the most heavily modified domains of histones and exert the greatest impact on chromatin structure. For example, the tails of histones H3 and H4 are located near the DNA entry/exit region (Figure 1). PTMs are more frequently found on the H3/H4 tails than the H2A/H2B tails, and many of them are associated with gene transcription and replication.^57^ As mentioned above, most of these histone modifications require metabolic intermediates as their substrates and are therefore affected by the metabolic state of the cell.

S‐adenosylmethionine (SAM) is a universal methyl donor for histone methylation. Methyltransferases transfer methyl groups from SAM to proteins and DNA, to produce S‐adenosyl‐L‐homocysteine (SAH) and methylated biomolecules.^16^, ^58^, ^59^ SAM is a metabolite derived from one‐carbon (1C) metabolism involving the folate and methionine cycles. Serine, glycine and threonine are the primary metabolic sources of 1C units. Serine is broken down to methyl‐tetrahydrofolate (THF) and glycine by serine hydroxymethyltransferase (SHMT). Glycine can be broken down again by the glycine cleavage system (GCS) to synthesize additional methyl‐THF. Threonine also supplies methyl‐THF, glycine and acetyl‐CoA to cells through a similar reaction mechanism.^58^, ^60^ In pluripotent mouse ESCs, threonine dehydrogenase (TDH)‐mediated threonine catabolism plays a key role in the regulation of histone methylation, as both threonine deprivation and TDH inhibition can reduce the SAM content in mESCs, thereby causing ESC differentiation.^60^, ^61^

Acetyl‐CoA is the two‐carbon (2C) metabolic substrate used to fuel histone acetylation.^16^, ^62^ During cellular metabolism, acetyl‐CoA is synthesized from pyruvate, citrate, acetate and β‐ketoacyl‐CoA, which are catabolism products of glucose, fatty acids and amino acids, respectively.^63^ Under culture conditions rich in carbohydrates, the mitochondrial pyruvate dehydrogenase (PDH) complex converts pyruvate to acetyl‐CoA.^64^, ^65^ In the mitochondria, citrate synthase combines the 2C portion of acetyl‐CoA with oxaloacetate to form citrate, which is then oxidized during the TCA cycle. Alternatively, citrate is transported into the cytoplasm, where ATP citrate lyase (ACL) cleaves citrate to regenerate acetyl‐CoA and oxaloacetate in the cytosol.^66^ Accumulation of cytosolic acetyl‐CoA can either promote the synthesis of lipids or promote the transportation of acetyl‐CoA into the nucleus. In the nucleus, acetyl‐CoA acetylates histones, resulting in gene activation.^67^ Therefore, the basal level of histone acetylation is highly dependent on the state of cellular catabolism.^67^

Studies have found that the early differentiation of pluripotent stem cells is accompanied by a decrease in acetyl‐CoA produced by glycolysis.^68^ In fact, it has been shown that glycolysis can improve the reprogramming efficiency of human and mouse fibroblasts.^69^, ^70^ In addition, studies have shown that ACL and cytosolic acetyl‐CoA play a crucial role in the acetylation of histones, with important implications for stem cell differentiation.^71^, ^72^, ^73^, ^74^ Thus, acetyl‐CoA availability can directly affect histone acetylation and serve as a metabolic signal to regulate cell fate decisions.

Histone (de)acetylation and (de)methylation are highly dynamic processes regulated not only by metabolic substrate availability, but also the metabolic enzymes themselves. Histone acetyltransferases (HATs) and histone deacetylases (HDACs) are two families of enzymes with opposing effects.^75^ HATs catalyse the transfer of acetyl groups from acetyl‐CoA to histone lysine residues, while HDACs remove acetyl groups from histone lysine residues. To date, 18 genes have been found to encode for HDACs in the mammalian genome. They are divided into four classes, each with different subcellular localizations and specificities. Class I HDACs include HDAC1, HDAC2, HDAC3 and HDAC8, which are mostly located in the nucleus. Class I HDACs often interact with different cofactors to form multiple repressive complexes such as Sin3A, NuRD and CoREST.^76^ Class II HDACs include HDAC4, HDAC5, HDAC6, HDAC7, HDAC9 and HDAC10, and they can shuttle between the nucleus and cytoplasm in response to different cell signals. Class III HDACs are comprised of the prominent SIRT1‐7 sirtuin proteins. Class IV has only one member, HDAC11.^77^ Class I, II and IV HDACs rely on Zn^2+^ to function, whereas Class III HDACs require NAD^+^ as a necessary cofactor. Amongst them, SIRT1, SIRT2, SIRT6 and SIRT7 are located in the nucleus and can deacetylate specific histone residues.

Similarly, histone methyltransferases (HMTs) and histone demethylases (HDMs) are also a pair of enzyme families with opposing effects. HMTs are a class of enzymes that mediate the methylation of histone lysine or arginine residues. These enzymes are highly selective for the histone residues they target and are divided into two types: arginine methyltransferases (PRMTs) and lysine methyltransferases (KMTs). To date, more than 50 human KMTs have been reported. Based on the catalytic domain, KMTs are further divided into two families: SET domain‐containing KMTs, which include the Su(var)3‐9, Polycomb and Trithorax proteins, and the non‐SET domain‐containing KMTs, such as DOT1‐like proteins.^78^, ^79^, ^80^ In contrast, lysine demethylases (KDMs) generally consist of two families: the KDM1 family of FAD‐dependent amine oxidases and the JmjC domain containing family of dioxygenases, which are enzymes that depend on Fe^2+^, ascorbate (vitamin C) and 2‐oxoglutarate (α‐ketoglutarate, α‐KG) as cofactors.^81^

Ascorbate and 2‐oxoglutarate have strong demethylation effects, which are used as metabolic agonists or cofactors of HDMs.^82^, ^83^, ^84^, ^85^ For example, in iPS reprogramming, the addition of ascorbate can enhance the expression of pluripotency genes, by removing H3K9me3 and H3K36me3 marks.^86^, ^87^ Studies in neural stem cells (NSCs) indicated that ascorbate can promote the removal of histone H3K9me3, H3K27me3 marks by JmjC domain‐containing HDMs, to upregulate a series of dopaminergic neuron‐specific genes. This increased the production of midbrain dopaminergic (mDA) neurons.^83^ Studies of 2‐oxoglutarate show that it can promote the self‐renewal of naive ESCs through increased histone H3K27me3 demethylation.^84^ Studies in mesenchymal stem cells (MSCs) derived from hESCs also showed that ascorbate and Fe^2+^ can act synergistically as cofactors to promote histone demethylation by JmjC demethylases, including the removal of H3K9me3, H3K36me3 and H3K27me1 marks, thereby promoting MSC fate specification, long‐term self‐renewal and senescence resistance.^88^

## 4 CHROMATIN‐MODIFYING DRUGS IN CELL FATE CONTROL

### 4.1 Histone‐modifying drugs

Small molecule drugs that regulate the activity of histone‐modifying enzymes, often as modified analogues of enzyme‐binding metabolites, also affect cell fate decisions. In particular, HDAC inhibitors (HDACi), HAT inhibitors (HATi), HMT inhibitors (HMTi) and HDM inhibitors (HDMi) have been found to play an important role in the regulation of cellular epigenetics and cell fate decisions. Below, we summarize some of the known effects of HDACi, HATi, HMTi and HDMi on cell fate determination.

#### 4.1.1 HDAC inhibitors

The most widely used HDACi can be generally divided into two categories based on their chemical structures and enzymatic activities: hydroxamates and fatty acids.

Hydroxamates were the first HDACi to be discovered. Because of their simple structures and powerful effects on HDACs as Zn^2+^ chelators, they were widely used and studied.^89^ Trichostatin A (TSA) is a pan‐HDAC inhibitor with the hydroxamate structure that was first isolated as a natural compound from *Streptomyces hygroscopicus* bacteria. It is composed of an aromatic cap group, a conjugated diene linker region and a hydroxamate tail which binds to the active site of HDACs in a non‐covalent manner and chelates Zn^2+^ ions away from the HDAC in a bidendate fashion.^90^ Treatment with TSA can increase the general level of histone acetylation in cells. Previous studies found that in mouse C2C12 myoblasts, TSA can increase the expression of early MRF transcription factors, Myf5 and MEF2, thereby promoting the differentiation of C2C12 cells.^91^ In addition, low concentrations of TSA can significantly promote the proliferation of MSCs and suppress their spontaneous osteogenic differentiation by regulating the acetylation of histone H3K9/K14, thus upregulating the mRNA expression of MSC multipotency and proliferative genes.^92^In terms of somatic cell nuclear transfer (SCNT), TSA also shows great potential. It improves the efficiency of development to full‐term state and favors the establishment of pluripotency of SCNT embryos by influencing histone acetylation status.^93^, ^94^

Panobinostat (LBH589) is another hydroxamate‐based HDACi. It acts on all Class I, Class II and Class IV HDACs, but it mainly acts on HDAC1/2/3/6.^89^ The drug has been shown to increase the levels of *CDKN1A* (p21) and induce excessive acetylation of H3 and H4.^95^, ^96^ By promoting the accumulation of acetylated histones, it can induce cell cycle arrest and apoptosis.^96^, ^97^ However, studies have also found that low doses of LBH589 can induce terminal differentiation and irreversible mitotic arrest, but not cell death, in committed osteogenic progenitors.^98^ Treatment with panobinostat upregulates osteogenic differentiation genes, including *RUNX2*, *ALPL*, *BMP4* and *SPP1*.

Belinostat (Beleodaq or PXD101) belongs to a new class of hydroxamate‐type HDACi, but it acts on the same targets as panobinostat.^89^ In MCF‐7 epithelial cells, belinostat inhibits cell proliferation by targeting the Wnt/β‐catenin and PKC pathways.^99^

Suberoylanilide hydroxamic acid (SAHA) is the first HDACi to be approved for clinical use on the market. It binds to the active site of Class I and Class II HDACs, with a predominant preference for Class I HDACs.^89^ Like TSA, it was found that SAHA treatment could reduce senescence and improve self‐renewal in MSCs.^100^ SAHA can also affect the differentiation potential of MSCs by regulating the inflammatory response.^101^ In synovium‐derived MSCs of the temporomandibular joint, IL‐1b‐mediated upregulation of IL‐6 and IL‐8 inhibits MSC cartilage formation potential. SAHA can inhibit IL‐1b‐mediated upregulation of IL‐6 and IL‐8 and regulate the repair function of MSCs. In human neural progenitor cells, SAHA treatment can activate brain‐derived neurotrophic factor (BDNF) mRNA expression, thereby promoting neural development and neurogenesis.^102^

Valproic acid (VPA) is a branched‐chain saturated fatty acid that comprises of a propyl substituent on a pentanoic acid stem. It is an inhibitor of Class I and IIa HDACs that has shown potent anti‐tumour effects. VPA functions as an HDACi most likely by binding to the catalytic centre of its target HDACs, thus blocking substrate access.^89^ Studies have found that low‐dose VPA treatment can promote pluripotency in ESCs.^103^ VPA induces a genome‐wide acetylation of histone H3K9 in ESCs, thereby changing the chromatin state and promoting pluripotency. In C2C12 myogenic progenitors or myoblasts, VPA has long‐term protective effects on myoblast survival, proliferation and differentiation by increasing histone acetylation.^104^ In addition, VPA can also induce neurogenic differentiation of human adipose tissue‐derived MSCs by activating canonical Wnt or non‐canonical Wnt signalling pathways.^105^

Sodium butyrate (NaBu or NaBt), a 4‐carbon (4C) fatty acid, can be synthesized and absorbed naturally after microbial metabolism in the colon. NaBu is a non‐competitive inhibitor of HDACs which acts mainly on HDAC2 and does not associate with the substrate‐binding site.^106^, ^107^ NaBu has an important effect on the maturation of oocytes and the expression of developmental genes. High concentrations of NaBu will hinder the meiosis of oocytes, but at low concentrations it can change the mRNA expression of developmental genes such as *Sox2* and *Oct4*, thereby improving the embryo quality.^106^ Previous studies have shown that NaBu can increase the expression of target genes by enhancing the acetylation of H3K9 and H4, thus promoting the differentiation of rat bone marrow‐derived MSCs into smooth muscle cells.^107^ NaBu can also promote the differentiation of satellite cells into myoblasts. This 4C fatty acid promotes the acetylation of genes that are conducive to muscle differentiation, such as *Mef2* and *MyoD*, thereby promoting myogenesis.^40^ In terms of iPS reprogramming, NaBu is more effective than TSA and VPA, and it can also promote the self‐renewal of ESCs and reduce their differentiation. However, ESCs are very sensitive to NaBu, and it will only promote self‐renewal within a narrow concentration range, inducing differentiation at higher concentrations.^108^ The physiochemical principles underlying these preferences and quantitative relationships remain unclear.

#### 4.1.2 HAT inhibitors

HATi are divided into three classes. Class I HATi are bisubstrate inhibitors, but they are not commonly used at present. Class II HATi are natural compounds, such as curcumin and garcinol. Class III HATi are synthetic compounds, which are more specific than natural compounds, such as C646.^109^

Garcinol is a natural compound isolated from *Garcinia indica*, a plant in the mangosteen family, and has been reported to inhibit p300 HAT via its binding to a non‐active site region of p300, resulting in a conformational change that decreases the binding affinities of p300 to acetyl‐CoA (uncompetitive inhibition) and histones (competitive inhibition).^110^, ^111^ Early studies have shown that garcinol can increase the ability of hematopoietic stem cells (HSCs) to expand in vitro and enhance their potential for homing to bone marrow by reducing the level of p53 acetylation.^112^, ^113^ It is worth noting that garcinol is also an effective neuroprotector, which enhances neuronal survival through the ERK signalling pathway.^114^

Curcumin is a natural compound isolated from different *Curcuma* species of plants and inhibits p300 HAT activity in the same way as garcinol.^109^ Studies have found that the addition of curcumin can inhibit the specificity of the cardiac lineage and the expression of cardiac muscle regulators in the early stages of cardiac differentiation. Early administration of curcumin inhibits ~ 94% of cardiomyogenesis by inhibiting the transcription and expression of *GATA4* and *MEF2C*.^115^ Curcumin also regulates NSC fate. It can induce neurogenesis, synapse generation and cell migration in adult brain‐derived NSCs in vitro.^116^

C646 is a synthetic p300 inhibitor, which shows the highest potency by competitively inhibiting acetyl‐CoA binding and non‐competitively inhibiting the binding of H4‐15 peptide substrate.^109^ In MSCs, C646 blocks p300 HAT activity effectively and delays aging by inhibiting the p53‐p21 signalling pathway.^117^ During the differentiation of hematopoietic cells, because C646 inhibits the interaction between p300 and *GATA1* and reduces *GATA1* acetylation and transcription activity, it significantly inhibits the erythroid differentiation mediated by EDAG.^118^

#### 4.1.3 HMT inhibitors

There is no clear classification of HMTi. Most of them are acting on HMTs through competitive or non‐competitive inhibition. The initially reported HMTi were analogues of the metabolite SAM, such as SAH and sinefungin,^119^ before more selective HMTi emerged.

GSK126 (GSK2816126) is a potent and highly selective Polycomb EZH2 inhibitor.^120^ GSK126 inhibits EZH2 through competitive inhibition of binding to SAM, which is required for histone methylation.^121^ Administration of GSK126 in hESCs can induce hESCs to differentiate into mesoderm and produce more MSCs by reducing H3K27me3.^121^ In leukaemia stem cells (LSCs), the addition of GSK126 can reduce the recruitment of H3K27me3 on the *PTEN* promoter, thereby increasing the expression of *PTEN* and ultimately reducing the number of LSCs.^122^ EPZ‐6438 (E7438/Tazemetostat) is also a potent EZH2 inhibitor with the same inhibitory mechanism as GSK126. In iPSC‐derived MSCs, EPZ‐6438 can effectively upregulate *PPARγ* gene expression and promote adipogenic differentiation through chromatin remodelling.^123^

BIX01294 (diazepin‐quinazolin‐amine derivative) is a competitive inhibitor against G9a, which can reduce G9a‐mediated H3K9 dimethylation, but not monomethylation. Unlike other HMT inhibitors, BIX01294 competes with the G9a substrate, instead of the G9a cofactor SAM.^120^ Earlier studies have shown that MSCs treated with BIX01294 showed increased expression of specific genes of various neuronal lineages along with a decrease in the level of H3K9me2, which effectively differentiated into neuron‐like cells.^124^ BIX01294 can also enhance the myocardial differentiation potential of BM‐MSCs.^125^ In this process, BIX01294 can enhance the proliferative capacity of myocardial progenitor cells without compromising their ability to function as myocardial progenitor cells during myocardial repair.^126^ Compared with BIX01294, UNC0638 is an inhibitor with lower toxicity and higher efficacy and specificity for G9a. UNC0638 can inhibit the lineage differentiation of human hematopoietic stem cells and progenitor cells (HSPCs) in vitro by inhibiting H3K9me2, so that HSPCs can retain their stem cell‐like phenotype and function after in vitro expansion.^127^

EPZ004777 and EPZ5676 are inhibitors of the H3K79 methyltransferase DOT1l, which act as competitive inhibitors of SAM.^120^ EPZ004777 can significantly improve reprogramming efficiency during the generation of mouse iPSCs. During this process, the level of H3K79me2 was significantly reduced, while the expression levels of *Oct4*, *Lin28*, *Sox2*, *Cdx2* and *Gata4*, which are related to pluripotency and early cell differentiation, increased on average.^128^ EPZ004777 can also cause increased ROS and autophagy activity in osteoclasts, leading to osteoclast differentiation.^129^ EPZ5676 has similar functions as EPZ004777.^120^, ^129^

#### 4.1.4 HDM inhibitors

Overexpression of HDMs and histone demethylation is a common theme in cancers, but loss of function occurs less frequently, thus motivating the search for HDM inhibitors to treat cancer cells.^81^ Studies have shown that neomorphic *IDH1/2* mutations can reduce α‐KG production and increase production of the α‐KG analogue 2‐hydroxyglutarate (2‐HG) instead, resulting in the inhibition of JmjC demethylases and genome‐wide changes in histone methylation.^85^ Many researchers have used the α‐KG dependence of the JmjC demethylases to design HDM inhibitors. There are two main skeletons for inhibitors of α‐KG‐dependent enzymes: N‐oxalylglycine (NOG), an α‐KG mimic that binds to the Fe^2+^ cofactor but is resistant to superoxide attack, and para‐2,4‐dicarboxylic acid (2,4‐PDCA), another α‐KG mimic that occupies the α‐KG binding site but which cannot complete catalysis.^130^ They affect cell fate by regulating both histone and DNA methylation.

GSK‐J4 is a specific inhibitor of Jmjd3, which can significantly inhibit the demethylation of H3K27.^130^ In Th17 cells, Jmjd3 directly binds to and reduces the level of H3K27me3 at the Rorc genomic site, thus upregulating the activity of the *Rorc* gene, which mainly encodes the Th17 transcription factors Rorγt and Th17 cytokine genes. Therefore, the administration of GSK‐J4 can significantly inhibit Th17 cell differentiation.^131^ Some studies have reported the relationship between GSK‐J4 and suppressed embryonic development. GSK‐J4 treatment also induces *GADD45B/G* expression in differentiated embryoid bodies, and this increased expression may be related to the regulation of cell proliferation, cell cycle and apoptosis.^132^ IOX1, a Jmjd2A inhibitor, can regulate the cell cycle of vascular smooth muscle cells (VSMCs) stimulated by angiotensin II (Ang II). Studies have shown that Jmjd2A levels are increased, while H3K9me3 levels are decreased in VSMCs stimulated by Ang II. The inhibition of Jmjd2A suppresses Ang II‐induced cell proliferation, migration and cell cycle progression, by inhibiting the expression of cyclin D1 and increasing the expression of *p21*.^133^ JIB‐04, a pan‐selective inhibitor of JmjC demethylases, can reduce the self‐renewal and stemness of cancer stem cells. JIB‐04 can significantly reduce the formation, growth, recurrence, invasion and migration of cancer stem cell tumour spheres by downregulating the expression of target genes related to cancer stem cell function regulated by Wnt/β‐catenin.^134^

## 5 DNA‐MODIFYING DRUGS

Beyond histones, the most critical part of chromatin would be the DNA double helix. According to the manner in which they bind to DNA, drugs that regulate DNA conformation can be divided into three types: intercalation, groove binding and covalent binding.

### 5.1 Intercalators

Intercalation refers to the insertion of molecules between DNA bases. Since intercalators do not break the DNA, they have a limited impact on DNA damage. According to their different molecular skeletons, intercalator drugs can be roughly divided into carbazole drugs, anthracycline drugs and acridine drugs.

Carbazole‐based drugs: curaxins are well‐known carbazole‐based drugs. The curaxin skeleton consists of electron‐withdrawing groups at positions 3 and 6 of the carbazole core, such as nitrosyl or carbonyl functional groups, and the aminoalkyl chain bound to N9 (Table 1). Computer simulations, circular dichroism and DNAse I footprinting all show that the carbazole group of CBL0137 is inserted between the bases of DNA,^135^, ^136^ which greatly increases the distance between the base pairs and unwinds the DNA double helix. The symmetrical side chains of the carbonyl group containing C3 and C6 extend into the major groove of DNA, while the N9 side chain is inserted into the minor groove of DNA. By using different concentrations of CBL0137, the dissociation constant (*K*_d_) of CBL0137‐DNA binding was found to be 40 ± 20 μM, where each nucleosome bound 2‐5 CBL0137 molecules. In addition, the insertion of CBL0137 into DNA changes it from B‐DNA to Z‐DNA.^136^ At the same time, it also destabilizes the nucleosome, separates the H2A‐H2B dimer^136^ and evicts histone H1 from the nucleosome.^51^ Hi‐C analysis further confirmed that CBL0137 can remodel the 3D structure of the genome, causing the TAD (topologically associating domain) boundary to be partially disrupted and the chromatin circle to disappear.^50^ The resultant decrease in distances between promoters and enhancers activates gene expression.^137^ CBL0137‐induced binding of FACT (facilitates chromatin transcription) and dissociation of CTCF (CCCTC‐binding factor) from insulator sites may be other reasons for these observed changes in 3D genome organization.^50^ In conclusion, CBL0137 neither inhibits topoisomerase II nor causes damage to DNA, but causes 3D chromatin remodelling after DNA intercalation. These changes result in a variety of cell signalling cascades, such as p53 and NF‐κB, leading to mitotic arrest or apoptosis of cancer cells.^50^, ^51^

**Table 1:** Carbazole‐based drugs

Carbazole: 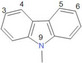		
Curaxins	C3 and C6	N9
CBLC000	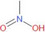	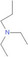
CBL0100	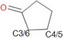	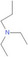
CBL0137	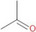	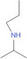

Anthracycline drugs: The basic structure of anthracycline drugs consists of a four‐ring unit connected to a sugar (see Table 2), which is a small planar molecule. Anthracycline drugs are relatively abundant, including doxorubicin, daunorubicin, epirubicin, idarubicin, aclacinomycin, valrubicin. Taking doxorubicin (Dox) as an example, its four‐membered planar ring is preferentially inserted between adjacent DNA base pairs to open the double helix, while the positively charged amino side chain on the sugar group interacts with the phosphate group on the DNA minor groove through electrostatic interactions to stabilize the doxorubicin‐DNA intercalation complex. In addition, the hydroxyl group on C9 forms a hydrogen bond with guanine, which also plays a role in stabilizing the doxorubicin‐DNA intercalation complex.^138^ Dox's influence on DNA conformation significantly changes the structure of nucleosomes and chromatin. Dox can induce the release of histones from nucleosomes in living cells independently of topoisomerase II.^139^, ^140^ It is possible that Dox's amino sugar competes with histone H4‐Arg residues for binding of the DNA minor groove.^139^ Another study showed that Dox can evict H2A from chromatin and evict H2B from the nucleus to the cytoplasm.^140^ Interestingly, Dox only binds to transcriptionally active regions^52^, ^139^, ^141^ and enhances nucleosome renewal in these regions, independently of the DNA damage response.^52^ Nevertheless, gene ontology analyses show that after Dox treatment of cells, genes related to the DNA damage response and cell cycle arrest are significantly upregulated.^52^ In yeast, genes involved in the oxidative stress response, DNA double‐strand break formation and autophagy are upregulated by Dox. Dox induction of DNA damage may be related to inhibiting the function of topoisomerase II, as well as producing oxidative stress,^142^ thereby inhibiting cell growth and promoting apoptosis. While this makes Dox a suitable anti‐cancer drug, it can also potentially cause serious damage to normal cells, especially in the heart.^139^

**Table 2:** Structure of anthracycline drugs

Anthracycline skeleton： 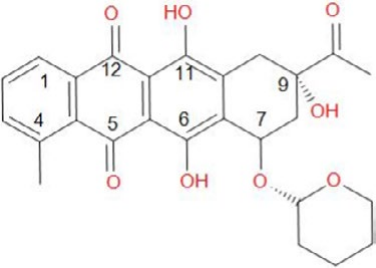			
Anthracycline	C4	C7	C9
Doxorubicin	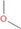	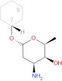	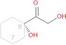
Daunorubicin	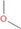	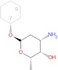	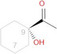
Epirubicin	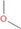	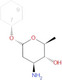	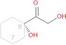
Idarubicin	‐H	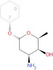	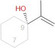

Acridines: In the natural or synthetic world of small molecules, drugs with an acridine group as the backbone are very abundant.^143^ Experiments using a viscometer system that measures changes in PM2 DNA hypervolume confirmed the hypothesis that the planar ternary acridine ring is inserted between the bases of DNA,^144^ which increases the distance between bases and changes DNA topology. Taking quinacrine as an example (see Table 3), besides the acridine ring inserted between the bases, its C9 nitrogen‐containing side chain can interact with the DNA minor groove to stabilize the binding of quinacrine to DNA.^145^ According to assays for the phosphorylation of histone H2A.X, quinacrine does not induce DNA double‐stranded breaks. However, quinacrine will activate p53, not via the phosphorylation of p53,^146^ but likely by loosening chromatin and capturing FACT to inhibit NF‐κB activity.^147^

**Table 3:** Acridines

Acridine： 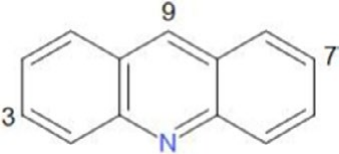			
Acridines	C3	C7	C9
9‐Ainoacridine （9AA）	‐H	‐H	‐NH2
Quinacrine (QC)	‐Cl	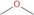	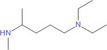

### 5.2 Groove binders

Groove binders non‐covalently bind to the DNA major and minor grooves through electrostatic interaction forces, van der Waals forces and hydrogen bonding. They are another class of drugs that do not damage DNA. Major grooves have multiple interaction sites to provide a relatively strong possibility of binding to drugs, together with an easily accessible channel for large molecules.^148^ Minor grooves have fewer joints and smaller sizes, but they are usually tension‐free, so they are also suitable targets of small molecule drugs.^149^ To match the structure of their binding site, the minor groove drugs are generally crescent‐shaped. The more typical minor groove drug distamycin A is rich in amide bonds, which easily form hydrogen bonds with bases. At the same time, its positive charges can interact with DNA phosphates through electrostatic forces, while the remaining structure is in a small trench.^148^ Unlike intercalation drugs, groove binders do not insert between base pairs and change the DNA topology less, thus limiting their potency as anti‐cancer drugs. The Hoechst 33 258 and DAPI dyes are examples of such non‐toxic minor groove binders. Nevertheless, distamycin A can still compete with histone H1 for DNA binding, causing H1 to be evicted from the nucleosome^150^, ^151^ and affecting the stability of the nucleosome. Furthermore, the addition of alkylating modification groups can increase the affinity and toxicity of groove binders.^152^ Existing natural major groove binders include pluramycins, aflatoxins, azinomycins, leinamycin, neocarzinostatin, ditercalinium.^143^ Compared with minor groove binders, the DNA alkylating effects of major groove binders are more pronounced. For example, aflatoxins and leinamycin can cause DNA breakage and damage through DNA alkylation.^149^

### 5.3 Covalent binders

Covalent binding of small molecules to DNA is often irreversible and therefore usually highly toxic. The alkylating agent is one of the universal DNA covalent binding agents. Alkylation refers to donating an alkyl group from a strong electrophilic compound via the formation of a covalent bond. Their cytotoxicity comes from the alkylation of DNA bases, leading to irreversible inhibition of basic DNA processes such as DNA replication and transcription.^152^ Well‐known alkylating agents include pyrrolobenzodiazepines (PBDs), platinum derivatives (cisplatin, carboplatin, oxaliplatine), oxazaphosphorines (cyclophosphamide, ifosfamide, trofosfamide), ethylene imines (mitomycin C, thiotepa, altretamine), nitrosoureas (MNU, BCNU, CCNU, nimustine), triazenes, hydrazines (dacarbazine, temozolomide, procarbazine), trabectedin,^150^, ^151^, ^152^ and they are all highly toxic.

### 5.4 Mechanistic Model

**Figure 2:**
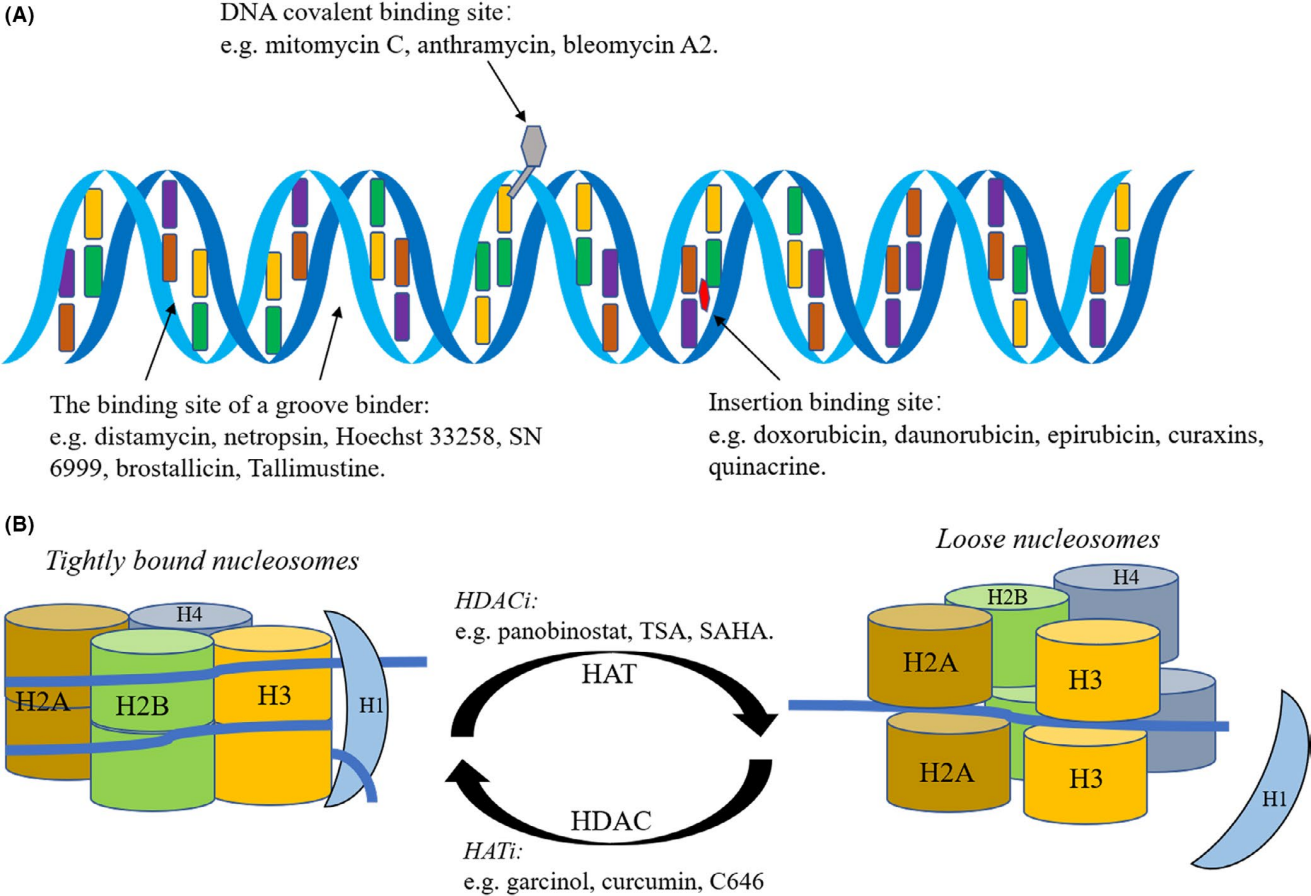
Summary of DNA and histone‐modifying drugs. A, DNA drugs mainly include three types: covalent binding agents, groove binders and insertion binders. Covalent binding agents, such as mitomycin, anthramycin and bleomycin, are usually very toxic. Groove binders can bind to the large and small grooves of DNA through non‐covalent bonding. Under normal circumstances, minor groove binders, such as distamycin A, do little damage to DNA. Insertion binders or intercalating agents are inserted between DNA bases and usually do not damage DNA. They can be roughly divided into anthracyclines (eg doxorubicin, daunorubicin, epirubicin), carbazoles (eg curaxins) and acridines (eg quinacrine) and could change B‐DNA to Z‐DNA. B, Histone drugs. Histone acetylation can be drugged via two families of proteins: HDACs and HATs. Amongst the HDAC inhibitors (HDACi), hydroxamates are the most widely used. Hydroxamates, such as TSA, panobinostat and SAHA, mainly function through the non‐covalent chelation of Zn^2+^ at the active site of HDACs. Together with the metabolic regulation of HATs, HDACi promote histone acetylation, eviction of histone H1 and nucleosome loosening. Amongst the HAT inhibitors (HATi), the most prominent are the natural compounds garcinol and curcumin, and the synthetic drug C646. Together with the metabolic regulation of HDACs, HATi promote histone deacetylation and tightly bound nucleosomes

Whether the targets of these small molecules are DNA or histones, the end result is often a change in chromatin conformation (Figure 2). Therefore, it is essential to understand the detailed mechanism of how changes in chromatin conformation are effected to regulate cell fate determination, to clarify and improve the effects of these drugs. Normally, chromatin is in a highly compressed state,^24^, ^153^ which helps to stabilize gene expression and prevent undirected changes in cell fate. However, condensed chromatin presents obstacles to directed changes in cell fate. Therefore, the processes of cell fate transitions and reprogramming will inevitably require chromatin remodelling. Below, we will synthesize extant knowledge of how this process is specifically achieved through both transcription factors and epigenetic regulation and hypothesize how drugs and metabolites could be involved.

**Figure 3:**
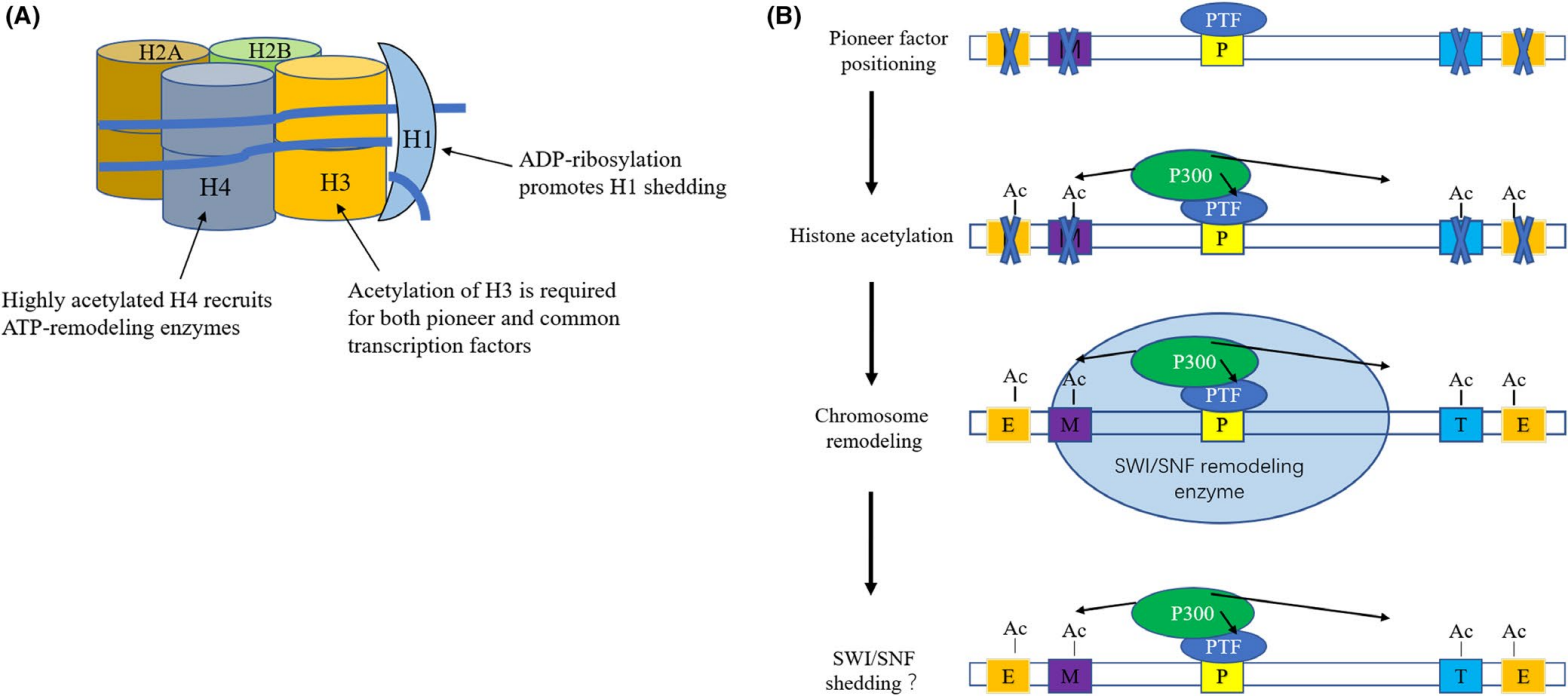
Mechanistic model of interactions between histone modifications and transcription factors during cell fate transitions. A, Several common histone modifications in the process of transcriptional regulation. ADP‐ribosylation promotes H1 eviction from nucleosomes, H3 acetylation promotes loosening of nucleosomes, while H4 acetylation can recruit ATP‐dependent remodelling enzymes for chromatin remodelling. Depending on the subtype, histone methylation can either loosen or tighten chromatin. B, During cell fate transitions, epigenetic histone modifications and pioneer transcription factors mutually influence each other to remodel chromatin organization and gene expression profiles. Pioneer transcription factors are a special type of transcription factor that can transiently bind to their DNA binding sites even if they reside in nucleosomes buried within condensed chromatin, but without any ostensible effects, unless further activated by histone acetylation and ATP‐dependent chromatin remodellers (eg SWI/SNF). The resultant changes in chromatin organization lead to different cell fates amongst isogenic cells. P, pioneer factor binding site; PTF: pioneer transcription factor; E, M, T: ordinary transcription factors; Ac: acetyl modification

In the early stages of cell fate transitions, a variety of epigenetic modifications regulated by metabolites and enzymes, including histone acetylation or ADP‐ribosylation or methylation, can regulate nucleosomes to provide either a loose or a tight chromatin environment for transcription factor binding (Figure 3A). Through this mechanism, cells are essentially able to sense the general nutrient and metabolic signals provided by the environment, prime their chromatin's nucleosomes to be tighter or looser and adjust their basal probabilities for cell fate transitions accordingly.

During this period, cell fate‐specific pioneer transcription factors might be expressed and transiently bind to their DNA targets (enhancers or promoters) regardless of chromatin condensation, but often with no ostensible effects (Figure 3B). However, if the nucleosomes are loosened based on the general metabolic state or the presence of chromatin‐modifying drugs, then pioneer factors can recruit chromatin remodellers to enter the nucleosomes and prompt the local opening of chromatin, thus providing a relaxed conformation for cell fate‐specific gene transcription in cooperation with other transcription factors downstream of other cell signalling pathways (Figure 3B), eventually leading to stable changes in specific cell fates.^154^, ^155^, ^156^ Although this process has been confirmed in myogenesis and neurogenesis mediated by MyoD and Ascl1 respectively,^157^, ^158^ there is still no consensus on the mechanism of how pioneer factors achieve initial binding, while the DNA is still hidden amongst nucleosomes. There are currently two plausible explanations that are distinct but not mutually exclusive: (a) high‐affinity binding of pioneer factors to nucleosomes. Pioneer factors usually possess a special DNA binding domain, such as Oct4 (POU domain), Sox2 (HMG box domain), FoxA (winged‐helix domain), which ensures that pioneer factors can transiently bind to the DNA side of the nucleosome^159^ and increase their residence time on the nucleosome.^160^ (b) Eviction of linker histone H1. For example, the structure of FoxA’s winged‐helix domain is highly similar to linker histone H1, and the eviction of H1 can facilitate the opening of condensed chromatin.^161^ Interestingly another metabolic modification—ADP‐ribosylation—can also reduce the affinity of H1 for DNA, to evict H1 and ready the nucleosomes for loosening.^162^ HMG proteins such as HMGA and HMGB likely play a role in the eviction of H1 linker histones after ADP‐ribosylation.^163^

Regardless of the specific mechanisms, after nucleosome loosening, pioneer factors can stably bind to the DNA, recruit HATs to increase the histone acetylation and recruit ATP‐dependent chromatin remodelling enzymes such as SWI/SNF to reorganize the chromatin. Different families of chromatin remodelling enzymes have a wide range of catalytic capabilities, including sliding histone octamers on DNA, changing nucleosome DNA conformation and changing histone octamer composition. For example, during myogenesis, MyoD recruits HATs to assist SWI/SNF localization and achieve regional remodelling of nucleosomal DNA,^164^ and further investigation showed that histone H4 hyperacetylation is necessary for this process.^165^

This mechanistic model provides a concise and qualitative explanation for the cell fate determination process. Small molecules and metabolites that regulate epigenetic modifications/enzymes prime the nucleosomes’ basal probabilities for transitions, based on nutrient metabolism and environmental conditions in general, while pioneer transcription factors downstream of cell signalling pathways drive cell fate transitions into specific directions. This generalized model remains incomplete in its details, particularly with regard to the code of cross‐interactions between histone modifications (ie the histone code),^12^, ^166^, ^167^ the combinations of pioneer transcription factors for each specific cell fate (ie the transcription factor code)^154^, ^155^, ^156^ and the specific details for how enhancers interact with various scaffold protein complexes and the lncRNA/eRNA/snRNA interactome to direct transcription (ie the ncRNA code).^168^ However, the biggest gap in this model lies in its qualitative nature. Without distilling the key forces underlying cell fate determination and the quantitative principles guiding these forces, it is extremely difficult to predict cell fate transitions based simply on a flood of molecular details on cells. Thus, in this age of single‐cell resolution ‘omics profiling’, there is an urgent need to identify and quantify the key quantitative relationships amongst chromatin‐modifying molecule concentrations, histone modifications, chromatin conformations, pioneer factor levels and their resultant cell fates, to facilitate the simulation of biophysical models that could allow us to predict and control cell fate transitions.

## 6 CONCLUSIONS AND PERSPECTIVES

By reviewing the corpus of research work on chromatin‐modifying metabolism and drugs in recent years, we can conclude that metabolites and drug analogues of metabolites play important roles in regulating cell fate determination. Certain trends are emerging, eg histone acetylation tends to be associated with stem cells and reprogramming efficiency, histone methylation/demethylation has complex effects on transcription and cell fate, while DNA drugs can also modulate higher‐order chromatin organization. However, the undeniable fact is that we still know too little to predict and control cell fate transitions. For instance, why does TSA promote MSC self‐renewal yet promote myoblast differentiation, while butyrate only promotes pluripotency in a very narrow concentration range? Although there are molecular explanations for these complex phenomena, we lack a unified model. Our new mechanistic model provides a new perspective on the role of chromatin modifications in promoting cell fate transitions, rather than promoting specific cell fates. Moving forward, it will be important to build upon this qualitative model to further explore the quantitative laws^169^ that govern the interactions between chromatin‐modifying molecules and cell fate transitions. If these metabolites and drug analogues can be used to precisely control the probabilities for cell fate transitions, they will have enormous potential in many fields beyond cancer, including anti‐aging and regenerative medicine.

## CONFLICT OF INTEREST

NS‐C. is a member of the Joint Steering Committee of the Ferring Institute of Reproductive Medicine, a research institute jointly funded by Ferring Pharmaceuticals and the Chinese Academy of Sciences to advance basic and translational research in reproductive medicine.

## AUTHOR CONTRIBUTIONS

ZY, YC, WC and N.S‐C. analysed, interpreted and wrote the manuscript. All authors read and approved the final manuscript.

## Data Availability

None available.
